# Bariatric Metabolic Surgery vs Glucagon-Like Peptide-1 Receptor Agonists and Mortality

**DOI:** 10.1001/jamanetworkopen.2024.15392

**Published:** 2024-06-07

**Authors:** Dror Dicker, Yael Wolff Sagy, Noga Ramot, Erez Battat, Philip Greenland, Ronen Arbel, Gil Lavie, Orna Reges

**Affiliations:** 1 Internal Medicine Department D and Obesity Clinic, Hasharon Hospital, Rabin Medical Center, Petah Tikva, Israel; 2 School of Medicine, Tel Aviv University, Tel Aviv, Israel; 3 Branch of Planning and Strategy, Clalit Health Services, Tel Aviv, Israel; 4 Department of Preventive Medicine, Northwestern University Feinberg School of Medicine, Chicago, Illinois; 5 Community Medical Services Division, Clalit Health Services, Tel Aviv, Israel; 6 Maximizing Health Outcomes Research Lab, Sapir College, Sderot, Israel; 7 Ruth and Bruce Rappaport Faculty of Medicine, Technion–Israel Institute of Technology, Haifa, Israel; 8 Department of Health Systems Management, Ariel University, Ariel, Israel

## Abstract

**Importance:**

Evidence regarding the relative effectiveness of bariatric metabolic surgery (BMS) and glucagon-like peptide-1 receptor agonists (GLP-1RAs) in reducing mortality and major adverse cardiovascular events (MACEs) is limited.

**Objective:**

To compare all-cause mortality and nonfatal MACEs associated with BMS vs GLP-1RAs for adults with obesity and diabetes and without known cardiovascular disease.

**Design, Setting, and Participants:**

This observational, retrospective cohort study was based on data obtained from the electronic medical records of Clalit Health Services (Clalit), the largest health care organization in Israel. The study included 6070 members aged 24 years or older, who had diabetes and obesity and no prior history of ischemic heart disease, ischemic stroke, or congestive heart failure. Patients who underwent BMS and patients who received GLP-1RAs from January 1, 2008, through December 31, 2021, were matched 1:1 by age, sex, and clinical characteristics. Follow-up ended December 31, 2022.

**Exposures:**

Initiation of BMS or GLP-1RAs.

**Main Outcomes and Measures:**

The primary outcome was all-cause mortality, assessed by multivariate Cox proportional hazards regression models. The secondary outcome was nonfatal MACEs, assessed by multivariate competing risk models.

**Results:**

The study included 3035 matched pairs of patients (total, 6070; mean [SD] age, 51.0 [9.5] years; 3938 women [64.9%]), who were followed up for a median of 6.8 years (IQR, 4.1-9.4 years). Among those with a diabetes duration of 10 years or less (2371 pairs), mortality was lower for those who underwent BMS than for those treated with GLP-1RAs (hazard ratio [HR], 0.38; 95% CI, 0.25-0.58). This association became nonsignificant when weight loss during the follow-up period was also included in the model (HR, 0.79; 95% CI, 0.43-1.48). Among patients with a duration of diabetes longer than 10 years (664 pairs), no survival advantage was demonstrated for BMS over GLP-1RA (HR, 0.65; 95% CI, 0.39-1.08). The risk for nonfatal MACEs did not differ between the treatment groups (HR, 0.74; 95% CI, 0.49-1.10 among patients with a diabetes duration of ≤10 years; HR, 1.21; 95% CI, 0.80-1.85 among patients with a diabetes duration of >10 years).

**Conclusions and Relevance:**

In this cohort study, BMS was associated with greater reduced mortality compared with first-generation GLP-1RAs among individuals with a diabetes duration of 10 years or less, mediated via greater weight loss. No differences in the risk for mortality were observed between the treatment modalities among individuals with a longer duration of diabetes, nor in the occurrence of nonfatal MACEs among all patients.

## Key Points

**Question:**

How are bariatric metabolic surgery (BMS) and glucagon-like peptide-1 receptor agonists (GLP-1RAs) associated with mortality and major adverse cardiovascular events (MACEs) among patients with obesity and diabetes?

**Findings:**

In this cohort study comprising 6070 patients, among individuals with a diabetes duration of 10 years or less, BMS was associated with a 62% reduction in mortality compared with GLP-1RAs, with weight reduction mediating the association. No difference was observed in the risk of mortality among those with a longer duration of diabetes, nor in the risk of MACEs among all patients.

**Meaning:**

This study suggests that BMS was associated with greater reduced mortality compared with GLP-1RAs among individuals with a diabetes duration of 10 years or less, mediated via greater weight loss.

## Teaser

This cohort study compares all-cause mortality and major adverse cardiovascular events associated with bariatric metabolic surgery vs glucagon-like peptide-1 receptor agonists for adults with obesity and diabetes.

## Introduction

Obesity is a chronic relapsing complicated disease characterized by adiposopathy, which impairs various body systems.^1^ The accumulating evidence of the associations of obesity with overall mortality, cardiovascular mortality, and a shorter life expectancy^2^ underscores the importance of implementing relevant interventions.

The association of bariatric metabolic surgery (BMS) with weight loss and reduced mortality and cardiovascular disease (CVD) are well established,^3^ notably among individuals with type 2 diabetes.^3,4,5^ Although the evidence supporting reduced mortality is derived primarily from observational studies^6,7,8,9^ rather than randomized clinical trials, a meta-analysis^3^ has provided compelling evidence for the advantages associated with BMS compared with usual care. International diabetes organizations have deemed BMS a key intervention for people with type 2 diabetes and obesity.^10^

Weight reduction and decreases in major cardiovascular events have also been demonstrated among patients with^11^ or without^12^ type 2 diabetes treated with glucagon-like peptide-1 receptor agonists (GLP-1RAs). Although both BMS and GLP-1RAs, separately, were shown to confer clinical advantages compared with conventional treatment, it is not clear how GLP-1RAs compare with BMS in reducing major adverse cardiovascular events (MACEs) and mortality. A recent study demonstrated lower risk for MACEs among individuals with diabetes who were treated with BMS compared with GLP-1RAs.^13^ However, that study did not differentiate between individuals with and individuals without a history of CVD, and 80.9% of patients underwent gastric bypass surgery. Considering the current evidence, the objective of this study was to compare mortality and cardiovascular outcomes of BMS vs treatment with GLP-1RA among adults with obesity and diabetes and without known CVD.

## Methods

### Study Design

This observational, retrospective cohort study was based on data obtained from the electronic medical records of Clalit Health Services (Clalit), the largest health care organization in Israel. Patients who underwent BMS or were treated with GLP-1RAs between January 1, 2008, and December 31, 2021, were matched and followed up for mortality and MACEs. Baseline characteristics were extracted as of the index date. Additional information about baseline measurements as of the index date can be found in the eMethods in Supplement 1. Follow-up ended on December 31, 2022. This study was approved by the Community Medical Services Division institutional review board and by data utilization committees of Clalit. Due to the retrospective design, the study was exempt from obtaining informed consent from the patients due to its retrospective design and because identifying information about patients was concealed. The study followed the Strengthening the Reporting of Observational Studies in Epidemiology (STROBE) reporting guideline for cohort studies.

### Study Population

The study included Clalit members aged 24 years or older with diabetes (diagnosed after 21 years of age) and a body mass index (BMI; calculated as weight in kilograms divided by height in meters squared) of 30 or greater, who underwent BMS or who were treated with GLP-1RAs from 2008 to 2021. Surgical patients were defined as having undergone laparoscopic banding, Roux-en-Y gastric bypass, or laparoscopic sleeve gastrectomy during these years. Patients were considered as treated with GLP-1RAs if they purchased first-generation GLP-1RAs (liraglutide, dulaglutide, exenatide, lixisenatide, or insulin degludec) for at least 6 months within a period of 12 consecutive months from 2008 to 2021. The index date was determined as the date of the BMS or of the first purchase of GLP-1RAs. For those who were treated with both BMS and GLP-1RAs from 2008 to 2021, the first treatment, with its index date, was considered. Exclusion criteria were as follows: oncologic diagnosis during the 2 years prior to the index date, end-stage kidney disease during the 2 years prior to the index date, pregnancy during the year prior to the index date, and history of ischemic heart disease, ischemic stroke, or congestive heart failure.

Eligible patients were matched 1:1 (1 patient who underwent BMS to 1 patient treated with GLP-1RAs) based on the following variables: sex, age group (in 5-year increments), BMI (in 5-unit increments), index date (in 2-year increments), and diabetes duration (the time from diagnosis until the index date, in 5-year increments).

### Outcomes

The primary outcome, all-cause mortality, was obtained from Ministry of Interior data. Secondary outcomes included nonfatal MACEs (myocardial infraction, stroke, or ischemic heart disease or the performance of percutaneous transluminal coronary angioplasty or coronary artery bypass graft during the follow-up period) and maximal and long-term changes in hemoglobin A_1c_ (HbA_1c_) concentration and in BMI. *Long-term change* refers to the latest time point available.

### Statistical Analysis

The characteristics of the individuals who underwent BMS and who received GLP-1RAs are described as mean (SD) values or median (IQR) values for continuous variables and as absolute numbers and percentages of individuals for categorical variables. Because outcomes associated with BMS may vary by the duration of diabetes,^14,15^ the associations of treatment type (BMS or GLP-1RAs) with the outcomes of interest were evaluated separately for patients with a diabetes duration of 10 years or less or more than 10 years. Kaplan-Meier curves and the log-rank test were used to compare the survival distributions between patients who underwent BMS and patients who were treated with GLP-1RAs.

Univariable and multivariable Cox proportional hazards regression models were used to assess the association of the treatment type with all-cause mortality. Multivariable models were adjusted for the following potential confounders as of the index date: diabetes duration (months), age (years), ethnicity (Jewish, non-Jewish, or unknown), BMI, HbA_1c_ concentration, socioeconomic status, a diagnosis of atrial fibrillation, a diagnosis of hyperlipidemia, a diagnosis of hypertension, smoking status, and using agents acting on the renin-angiotensin system, lipid-modifying agents, insulin, sodium-glucose cotransporter-2 inhibitors, or other blood glucose–lowering drugs. The Schoenfeld global test was used to test the proportional hazards assumption for all the variables. To assess the association between the type of treatment and nonfatal MACEs, Fine-Gray competing risks regression models were used, which accounted for the effect of competing risks of death. Variance inflation factors were calculated to test for multicollinearity between all the covariates included in the adjusted models.

Patients were censored from the analyses according to the earliest of the following: a change in treatment, discontinuation of membership with Clalit, occurrence of the event of interest, reaching 12 years’ follow-up, or December 31, 2022. A change in treatment was considered as initiation of treatment with GLP-1RAs for patients who underwent BMS or the performance of BMS among patients who had received GLP-1RAs. To assess whether mortality and MACEs associated with BMS vary by diabetes duration, the multiplicative interaction term of treatment type and diabetes duration was incorporated into the Cox proportional hazards regression model and the Fine-Gray competing risks regression model, respectively. When a significant association between treatment type and an outcome of interest was identified, the potential mediation effects of weight loss (measured by percentages of the maximal change in BMI during the follow-up period) and improvement in HbA_1c_ levels (measured by the maximal change in HbA_1c_ concentration during the follow-up period) were assessed. In each mediation analysis, the association between treatment type and the potential mediator (using linear models) as well as the association between the potential mediator and the outcome of interest (using Cox models) were examined. Additionally, the inclusion of the potential mediator in the adjusted model was evaluated for the association between treatment type and an outcome of interest.

To prevent the inclusion of individuals with preexisting oncologic disease detected within the first 2 years of follow-up, a sensitivity analysis excluding such individuals was performed. Independent samples *t* tests were used to estimate the statistical significance of the absolute difference in changes in HbA_1c_ concentration and BMI (maximal and long term) between patients who underwent BMS and patients who were treated with GLP-1RAs. Analyses were performed with R statistical software, version 4.0.1 (R Project for Statistical Computing). All *P* values were from 2-sided tests, and results were deemed statistically significant at *P* < .05.

## Results

### Patient Selection

From 2008 to 2021, among members of Clalit aged 24 years or older who had diabetes and a BMI of 30 or greater, 5304 patients underwent BMS, and 47 427 patients initiated treatment with GLP-1RAs. Of those patients, 1042 patients who underwent BMS and 14 773 patients who initiated GLP-1RAs were excluded due to active cancer, end-stage kidney disease, pregnancy, or a history of ischemic heart disease, ischemic stroke, or congestive heart failure.

After the matching procedure, 1227 patients who underwent BMS and 29 619 patients who initiated GLP-1RAs were excluded due to no suitable match. A total of 3035 matched pairs were included in the analyses (eFigure in Supplement 1).

### Patient Population

The study population included 6070 individuals (mean [SD] age, 51.0 [9.5] years; 3938 women [64.9%] and 2132 men [35.1%]). The mean (SD) BMI at the index date was 41.3 (5.1). Compared with patients who underwent BMS, those who were treated with GLP-1RAs had higher mean (SD) HbA_1c_ levels (9.1% [1.7%] vs 7.5% [1.5%]; to convert to proportion of total hemoglobin, multiply by 0.01), triglyceride levels (217.5 [156.0] mg/dL vs 199.5 [190.0] mg/dL; to convert to millimoles per liter, multiply by 0.0113), and fasting plasma glucose levels (197.2 [67.0] mg/dL vs 151.8 [55.2] mg/dL; to convert to millimoles per liter, multiply by 0.0555) (Table 1). Patients who underwent BMS were less likely to have hyperlipidemia and hypertension and used less insulin and fewer agents acting on the renin-angiotensin system, sodium-glucose cotransporter-2 inhibitors, and other blood glucose–lowering drugs compared with individuals who were treated with GLP-1RAs.

**Table 1.** Characteristics of Matched Patients Who Underwent BMS or Received GLP-1RAs

Characteristic	Patients, No. (%)		*P* value
	BMS (n = 3035)	GLP-1RAs (n = 3035)	
Type of BMS			
Laparoscopic banding	376 (12.4)	NA	NA
Gastric bypass	1408 (46.4)	NA	
Sleeve gastrectomy	1251 (41.2)	NA	
Reoperations			
Within the first year	18 (0.6)	NA	NA
After the first year of follow-up	82 (2.7)	NA	
GLP-1RA			
Liraglutide	NA	1879 (61.9)	NA
Dulaglutide	NA	642 (21.2)	
Exenatide	NA	412 (13.6)	
Lixisenatide	NA	43 (1.4)	
Insulin degludec and liraglutide	NA	50 (1.6)	
Insulin degludec and lixisenatide	NA	9 (0.3)	
Sex			
Male	1066 (35.1)	1066 (35.1)	>.99
Female	1969 (64.9)	1969 (64.9)	
Age, y			
Mean (SD)	50.9 (9.52)	51.1 (9.54)	.06
Median (IQR)	51.0 (44.0-58.0)	51.0 (44.0-58.0)	
Age group, y			
24 to <40	376 (12.4)	376 (12.4)	
40 to <60	2043 (67.3)	2043 (67.3)	
≥60	616 (20.3)	616 (20.3)	
Ethnicity			
Jewish	2305 (75.9)	1967 (64.8)	<.001
Non-Jewish	727 (24.0)	1068 (35.2)	
Unknown	3 (0.1)	0	
SES			
Low	589 (19.4)	869 (28.6)	<.001
Medium	1851 (61.0)	1564 (51.5)	
High	448 (14.8)	430 (14.2)	
Unknown	147 (4.8)	172 (5.7)	
Immigration status			
Born in Israel	2186 (72.0)	2175 (71.7)	.70
Immigrant to Israel	848 (27.9)	860 (28.3)	
Unknown	1 (0.03)	0	
Peripherality level^a^			
Mean (SD)	6.2 (2.1)	6.4 (2.0)	<.001
Median (IQR)	6.0 (5.0-8.0)	6.0 (5.0-8.0)	
Diagnosis of comorbidities			
Atrial fibrillation	60 (2.0)	53 (1.7)	.05
Hyperlipidemia	2330 (76.8)	2458 (81.0)	<.001
Hypertension	1637 (53.9)	1763 (58.1)	<.001
Diabetes duration, y			
Mean (SD)	6.2 (5.8)	6.7 (5.6)	<.001
Median (IQR)	4.8 (1.5-9.4)	4.9 (2.8-9.5)	
Hemoglobin A_1c_, %			
Mean (SD)	7.5 (1.5)	9.1 (1.7)	<.001
Median (IQR)	7.1 (6.5-8.1)	8.8 (7.9-10.0)	
<6.0	271 (8.9)	28 (0.9)	
6.0 to <6.5	476 (15.7)	50 (1.6)	
6.5 to <7.0	616 (20.3)	110 (3.6)	
7.0 to <7.5	479 (15.8)	165 (5.4)	
7.5 to <8.0	319 (10.5)	438 (14.4)	
8.0 to <8.5	216 (7.1)	466 (15.4)	
8.5 to <9.0	162 (5.3)	387 (12.8)	
≥9.0	461 (15.2)	1374 (45.3)	
Unknown	35 (1.2)	17 (0.6)	
Fasting plasma glucose, mg/dL			
Mean (SD)	151.8 (55.2)	197.2 (67.0)	<.001
Median (IQR)	137.0 (115.0-172.0)	184.0 (150.0-233.0)	
Unknown	11 (0.4)	23 (0.8)	
Total cholesterol, mg/dL			
Mean (SD)	180.9 (41.2)	174.4 (40.1)	<.001
Median (IQR)	176.9 (153.0-204.0)	168.0 (147.4-198.0)	
Unknown	13 (0.4)	13 (0.4)	
HDL cholesterol, mg/dL			
Mean (SD)	43.6 (11.4)	41.7 (10.0)	<.001
Median (IQR)	42.0 (36.0-50.0)	41.0 (35.0-41.7)	
Unknown	14 (0.5)	13 (0.4)	
LDL cholesterol, mg/dL			
Mean (SD)	119.6 (39.3)	114.5 (41.2)	<.001
Median (IQR)	116.0 (92.0-144.0)	109.1 (85.4-138.0)	
Unknown	192 (6.3)	237 (7.8)	
Triglycerides, mg/dL			
Mean (SD)	199.5 (190.0)	217.5 (156.0)	<.001
Median (IQR)	167.0 (124.0-233.0)	181.0 (134.0-248.0)	
Unknown	13 (0.4)	12 (0.4)	
Systolic blood pressure, mm Hg			
Mean (SD)	129.9 (13.4)	130.4 (13.8)	.30
Median (IQR)	130.0 (120.0-138.0)	130.0 (121.0-138.0)	
Unknown	31 (1.0)	22 (0.7)	
Diastolic blood pressure, mm Hg			
Mean (SD)	77.9 (9.1)	78.3 (9.0)	.04
Median (IQR)	79.0 (71.0-83.0)	80.0 (72.0-84.0)	
Unknown	31 (1.0)	22.0 (0.7)	
BMI			
Mean (SD)	41.5 (5.1)	41.2 (5.2)	.03
Median (IQR)	40.6 (37.9-44.2)	40.6 (37.2-44.1)	
30 to <35	151 (5.0)	151 (5.0)	
35 to <40	1148 (37.8)	1148 (37.8)	
40 to <45	1104 (36.4)	1104 (36.4)	
45 to <50	441 (14.5)	441 (14.5)	
≥50	191 (6.3)	191 (6.3)	
Smoking status			
Nonsmoker	1641 (54.1)	1767 (58.2)	<.001
Former	555 (18.3)	445 (14.7)	
Current	554 (18.3)	481 (15.8)	
Unknown	285 (9.4)	342 (11.3)	
Pharmaceutical treatment			
β-Blocking agents	646 (21.3)	755 (24.9)	.08
Calcium channel blockers	550 (18.1)	554 (18.3)	.30
Agents acting on the renin-angiotensin system	1690 (55.7)	2006 (66.1)	<.001
Lipid-modifying agents	2014 (66.4)	2340 (77.1)	<.001
Insulins	568 (18.7)	1046 (34.5)	
Blood glucose–lowering drugs, excluding insulins	2320 (76.4)	2905 (95.7)	
Antidepressants	552 (18.2)	569 (18.7)	.30
Medications for weight reduction	73 (2.4)	71 (2.3)	.50
Sodium-glucose cotransporter 2 inhibitors	49 (1.6)	127 (4.2)	<.001

Abbreviations: BMI, body mass index (calculated as weight in kilograms divided by height in meters squared); BMS, bariatric metabolic surgery; GLP-1RA, glucagon-like peptide-1 receptor agonist; HDL, high-density lipoprotein; LDL, low-density lipoprotein; NA, not applicable; SES, socioeconomic status.

SI conversion factors: To convert hemoglobin A_1c_ to proportion of total hemoglobin, multiply by 0.01; glucose to millimoles per liter, multiply by 0.0555; total cholesterol, HDL cholesterol, and LDL cholesterol to millimoles per liter, multiply by 0.0259; and triglycerides to millimoles per liter, multiply by 0.0113.

^a^ Peripherality level indicates accessibility and proximity to the Tel Aviv district (0 indicates the minimum score and 10 indicates the maximum score).

The median follow-up time was 6.8 years (IQR, 4.1-9.4 years); the maximum follow-up time was 12 years. A change in treatment, resulting in discontinuation of follow-up, was observed among 352 of 3035 patients who underwent BMS (11.6%) and who had also initiated treatment with GLP-1RAs during the follow-up period. In addition, 252 of 3035 patients taking GLP-1RAs (8.3%) underwent BMS during follow-up.

### Stratification by Duration of Diabetes

The duration of diabetes was defined as the time elapsed from the diagnosis of diabetes until the initiation of either treatment with GLP-1RAs or BMS. The duration of diabetes was shown to interact with mortality associated with GLP-1RA vs BMS (*P* = .03 for the interaction treatment type × diabetes duration). Thus, all results are reported separately for patients with a diabetes duration of 10 years or less or more than 10 years at index date.

The cohort comprised 2371 pairs of patients with a diabetes duration of 10 years or less (median age, 50 years [IQR, 43-56 years]) and 664 pairs of patients with a diabetes duration of longer than 10 years (median age, 57 years [IQR, 50-62 years]). No interaction was observed between age at baseline and type of treatment in their association with mortality.

### Association Between Type of Treatment and All-Cause Mortality

**Figure 1.**
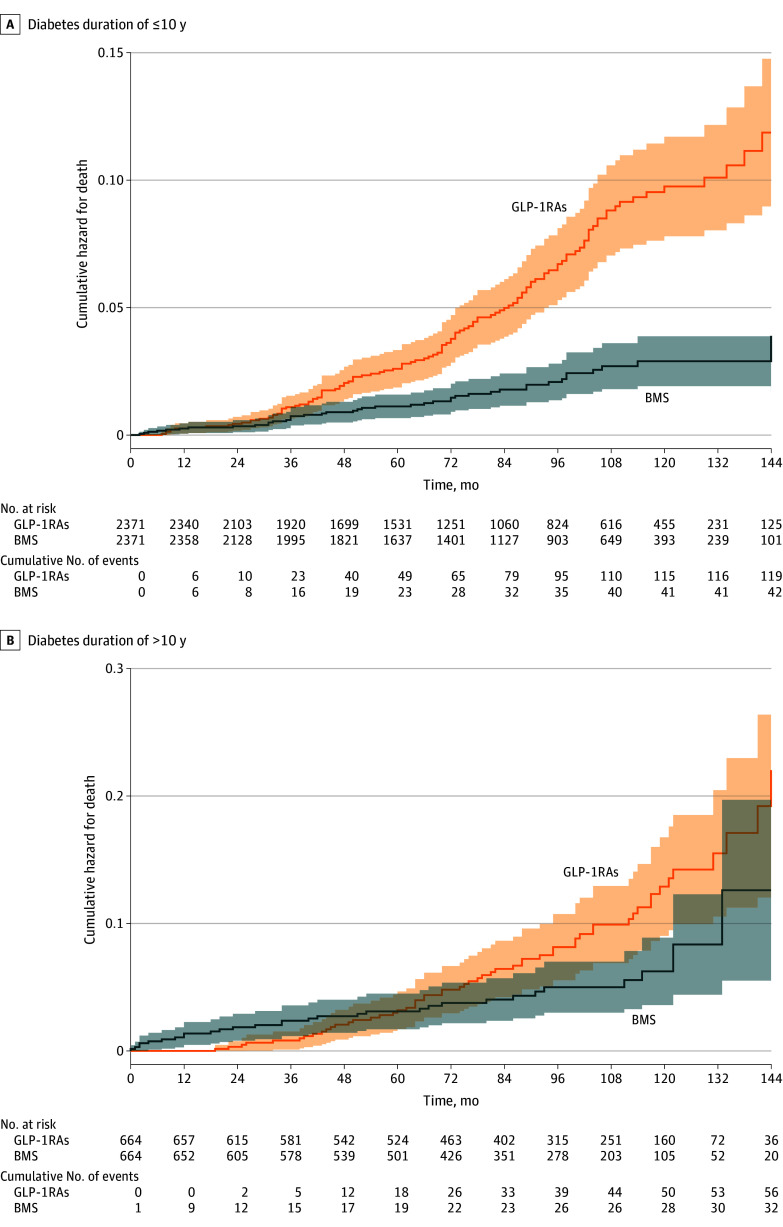
Cumulative Hazard for Death A, Bariatric metabolic surgery (BMS) vs treatment with glucagon-like peptide-1 receptor agonists (GLP-1RAs) among patients with a diabetes duration of 10 years or less. B, BMS vs treatment with GLP-1RAs among patients with a diabetes duration of more than 10 years. Shaded regions correspond to the 95% CIs for the cumulative hazard curves.

Among patients with a diabetes duration of 10 years or less, 42 of those who underwent BMS (266 per 100 000 person-years) and 119 of those treated with GLP-1RAs (785 per 100 000 person-years) died during the follow-up period (Table 2). The Kaplan-Meier estimated mortality curves demonstrated significantly lower mortality among the patients who underwent BMS than among those who were treated with GLP-1RAs (*P* < .001 by log-rank test) (Figure 1A).

**Table 2.** Differences in Outcomes Between Patients Who Underwent BMS and Matched Patients Who Were Treated With GLP-1RAs

Outcome	Diabetes diagnosis ≤10 y (n = 4742)			Diabetes diagnosis >10 y (n = 1328)		
	BMS (n=2371)	GLP-1RAs (n=2371)	*P* value	BMS (n=664)	GLP-1RAs (n=664)	*P* value
Duration of follow-up, y						
Mean (SD)	6.7 (3.2)	6.4 (3.3)	.005	6.9 (3.0)	7.4 (3.1)	.005
All-cause mortality, No. (%)	42 (1.8)	119 (5.0)	<.001	32 (4.8)	56 (8.4)	.03
Nonfatal MACEs, No. (%)	69 (2.9)	109 (4.6)	.002	51 (7.7)	58 (8.7)	.48
BMI at index date						
Mean (SD)	41.7 (5.0)	41.4 (5.2)	.11	40.7 (5.1)	40.4 (5.1)	.22
BMI maximal change, mean (SD)^a^						
Absolute change	−13.1 (4.8)	−5.3 (4.1)	<.001	−11.8 (4.9)	−5.2 (3.7)	<.001
Change from baseline, %	−31.4	−12.8		−29.0	−12.9	
BMI long-term change, mean (SD)^b^						
Absolute change	−10.1 (5.4)	−3.1 (4.3)	<.001	−8.9 (5.1)	−2.9 (4.2)	<.001
Change from baseline, %	−24.2	−7.5		−21.9	−7.2	
Hemoglobin A_1c_ level at index date, %						
Mean (SD)	7.3 (1.4)	9.0 (1.7)	<.001	8.0 (1.7)	9.3 (1.6)	<.001
Hemoglobin A_1c_ level maximal change, mean (SD), %^a^						
Absolute change, %	−1.8 (1.4)	−2.5 (1.7)	<.001	−2.0 (1.5)	−2.5 (1.6)	<.001
Hemoglobin A_1c_ long-term change, mean (SD), %^b^						
Absolute change, %	−1.2 (1.5)	−1.3 (2.1)	.20	−1.0 (1.8)	−1.5 (1.9)	<.001

Abbreviations: BMI, body mass index (calculated as weight in kilograms divided by height in meters squared); BMS, bariatric metabolic surgery; GLP-1RA, glucagon-like peptide-1 receptor agonist; MACE, major adverse cardiovascular event.

SI conversion factor: To convert hemoglobin A1c to proportion of total hemoglobin, multiply by 0.01.

^a^ Maximal change was calculated as the difference between the level at the index date and the lowest level achieved during the follow-up period.

^b^ Long-term change was calculated as the difference between the level at the index date and the most recent level achieved during the follow-up period.

The adjusted Cox proportional hazards regression model demonstrated a lower risk for death among those who underwent BMS compared with those who were treated with GLP-1RAs (hazard ratio [HR], 0.38; 95% CI, 0.25-0.58) (Table 3). The association was preserved when the maximal change in HbA_1c_ concentration during follow-up was included in the model (HR, 0.43; 95% CI, 0.27-0.99) but disappeared once the percentages of the maximal change in BMI level were also introduced into the model (HR, 0.79; 95% CI, 0.43-1.48). The mediation analysis with weight loss as a mediator revealed a significant association between treatment type and weight loss (estimate, −18.42; *P* < .01), as well as between weight loss and mortality (HR, 1.05; 95% CI, 1.02-1.08).

**Table 3.** Associations Between Treatment Type and Outcomes According to the Duration of Diabetes

Outcome	Diabetes duration ≤10 y (n = 4742)	Diabetes duration >10 y (n = 1328)
All-cause mortality, No. (%)	161 (3.4)	88 (6.6)
Nonfatal MACEs, No. (%)	178 (3.8)	109 (8.2)
BMS vs treatment with GLP-1RAs, HR (95% CI)		
All-cause mortality		
Unadjusted	0.33 (0.23-0.47)	0.65 (0.42-0.995)
Adjusted^a^^,^^b^	0.38 (0.25-0.58)	0.65 (0.39-1.08)
Adjusted, including HbA_1c_ level maximal change	0.43 (0.27-0.99)	NA
Adjusted, including BMI level maximal change	0.79 (0.43-1.48)	NA
Nonfatal MACE		
Unadjusted	0.61 (0.46-0.82)	0.89 (0.62-1.30)
Adjusted^a^	0.74 (0.49-1.10)	1.21 (0.80-1.85)

Abbreviations: BMI, body mass index (calculated as weight in kilograms divided by height in meters squared); BMS, bariatric metabolic surgery; GLP-1RA, glucagon-like peptide-1 receptor agonist; HbA_1c_, hemoglobin A_1c_; HR, hazard ratio; MACE, major adverse cardiovascular event; NA, not applicable.

^a^ The models were adjusted for the following variables as of the index date: diabetes duration (months), age (years), ethnicity (Jewish, non-Jewish, unknown), BMI, HbA_1c_ level, socioeconomic status, diagnosis of atrial fibrillation, diagnosis of hyperlipidemia, diagnosis of hypertension, smoking status (nonsmoker, former, current, unknown), and using agents acting on the renin-angiotensin system, lipid-modifying agents, insulin, sodium-glucose cotransporter-2 inhibitors, and other blood glucose–lowering drugs. No multicollinearity was observed between variables included in the model (variance inflation factors <1.7). The models met the proportional hazards assumption.

^b^ Schonfeld global test for mortality model, for patients with a diabetes duration of less than 10 years, 0.93; for patients with a diabetes duration of longer than 10 years, 0.59.

Similar results were observed when individuals who received a diagnosis of cancer in the first 2 years of follow-up were excluded from the analysis (eResults and eTable in Supplement 1). Among the patients with a diabetes duration of longer than 10 years, 32 of those who underwent BMS (695 per 100 000 person-years) and 56 of those treated with GLP-1RAs (1139 per 100 000 person-years) died during the follow-up period (Table 2). The Kaplan-Meier estimated mortality curves demonstrated significantly lower mortality among the patients who underwent BMS compared with those who were treated with GLP-1RAs (*P* = .047 by log-rank test) (Figure 1B). However, the adjusted Cox proportional hazards regression model demonstrated no statistically significant difference between groups (HR, 0.65; 95% CI, 0.39-1.08) (Table 3).

### Association Between Type of Treatment and Nonfatal MACEs

**Figure 2.**
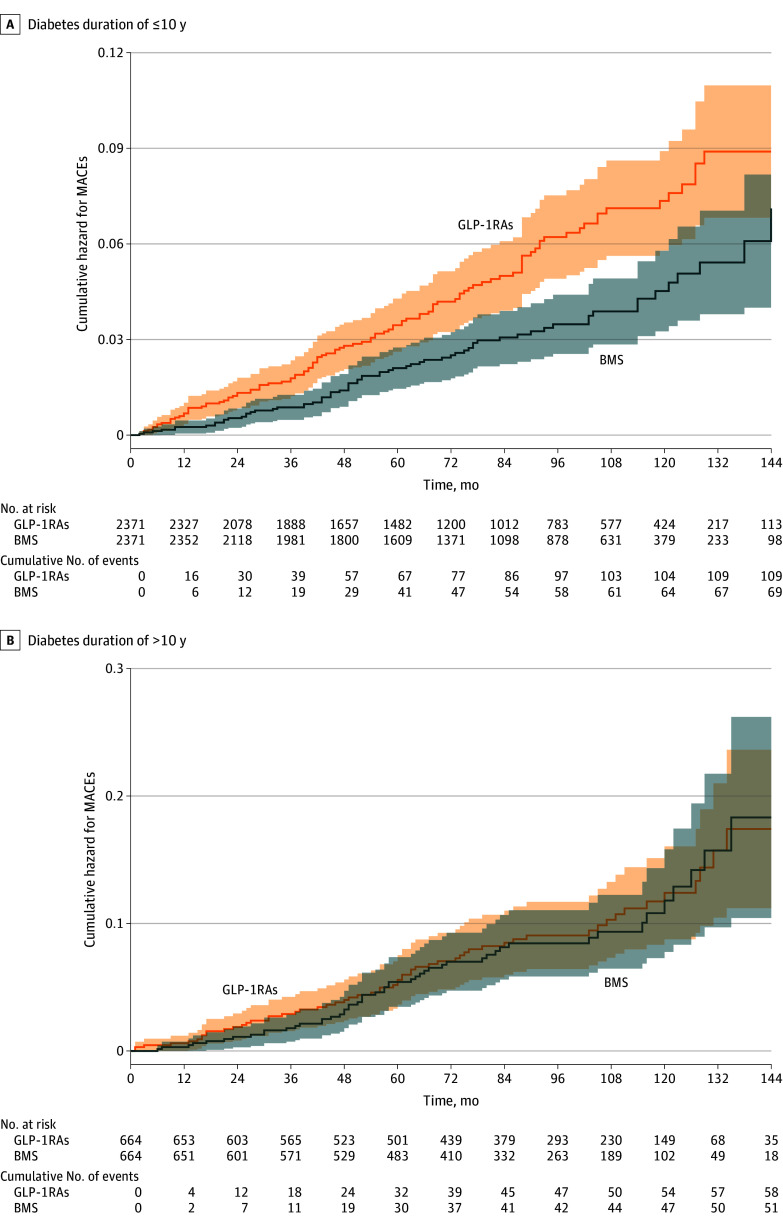
Cumulative Hazard for Nonfatal Major Adverse Cardiovascular Events (MACEs) A, Bariatric metabolic surgery (BMS) vs treatment with glucagon-like peptide-1 receptor agonists (GLP-1RAs) among patients with a diabetes duration of 10 years or less. B, BMS vs treatment with GLP-1RAs among patients with a diabetes duration of more than 10 years. Shaded regions correspond to the 95% CIs for the cumulative hazard curves.

Among patients with a diabetes duration of 10 years or less, 69 of 2371 patients who underwent BMS (443 per 100 000 person-years) and 109 of the 2371 patients treated with GLP-1RAs (739 per 100 000 person-years) had nonfatal MACEs during the follow-up period (Table 2). The Kaplan-Meier estimated mortality curves demonstrated a significantly lower incidence of nonfatal MACEs among the patients who underwent BMS than among those who were treated with GLP-1RAs (*P* < .001 by log-rank test) (Figure 2A). However, a competing risks regression model adjusted for potential confounders demonstrated no statistically significant difference in the risk for nonfatal MACEs between groups (HR, 0.74; 95% CI, 0.49-1.10) (Table 3).

Among patients with a diabetes duration of longer than 10 years, 51 of the 664 who underwent BMS (1134 per 100 000 person-years) and 58 of 664 patients treated with GLP-1RAs (1226 per 100 000 person-years) had nonfatal MACEs during the follow-up period (Table 2). The Kaplan-Meier model showed no significant differences in nonfatal MACEs between groups (*P* = .80 by log-rank test) (Figure 2A). The adjusted competing risks regression models demonstrated no association between treatment type and the risk for nonfatal MACEs (HR, 1.21; 95% CI, 0.80-1.85) (Table 3).

### Changes in HbA_1c_ Concentration and BMI

Among individuals with a diabetes durations of 10 years or less, the maximal change in BMI was greater among those who underwent BMS (mean [SD] absolute change, –13.1 [4.8]; relative mean change, −31.4%) than among those treated with GLP-1RAs (mean [SD] absolute change, –5.3 [4.1]; relative mean change, −12.8%) (Table 2). Similarly, the long-term BMI decrease was greater among patients who underwent BMS (mean [SD] absolute change, –10.1 [5.4]; relative mean change, −24.2%) than among patients treated with GLP-1RAs (mean [SD] absolute change, –3.1 [4.3]; relative mean changes, −7.5%). Similar results in BMI changes were observed among patients with diabetes duration of longer 10 years.

As for glycemic control, among individuals with 10 years or less of diabetes, the maximal change in HbA_1c_ concentration was lower among those who underwent BMS (mean [SD] absolute change, −1.8% [1.4%]) than those treated with GLP-1RAs (mean [SD] absolute change, −2.5% [1.7%]) (Table 2). The long-term change in HbA_1c_ concentration was similar between those who underwent BMS (mean [SD] absolute change, −1.2% [1.5%]) and those who treated with GLP-1RAs (mean [SD] absolute change, −1.3% [2.1%]). Among individuals with more than 10 years of diabetes, both the maximal change and the long-term change were lower among those who underwent BMS (mean [SD] maximal change, −2.0% [1.5%]; long-term change, −1.0% [1.8%]) than those who were treated with GLP-1RAs (mean [SD] maximal change, −2.5% [1.6%]; long-term change, −1.5% [1.9%]).

## Discussion

In this retrospective cohort study, among individuals with obesity and a diabetes duration of 10 years or less, but with no prior history of ischemic heart disease, ischemic stroke, or congestive heart failure, BMS was associated with a 62% reduction (HR, 0.38; 95% CI, 0.25-0.58) in mortality compared with GLP-1RAs, after adjustment for potential confounders. This association was mediated by weight reduction. No difference was observed in the risk of mortality among those with a longer duration of diabetes, nor in the risk of MACEs among all patients.

Survival advantages compared with standard care among individuals with diabetes were previously shown for both BMS^16^ and treatment with GLP-1RAs.^17^ In their meta-analysis, van Veldhuisen et al^16^ demonstrated a 45% reduction in all-cause mortality among individuals who underwent BMS compared with persons with obesity who were treated without surgery, although only 1 study^18^ differentiated between individuals with diabetes and individuals without diabetes. In a meta-analysis by Syn et al,^3^ lower rates of all-cause mortality were demonstrated among patients treated with surgery compared with patients treated without surgery; the reduction in risk was greater for individuals with diabetes than for individuals without diabetes (59.1% vs 29.6%). As several studies demonstrated a more pronounced survival advantage associated with BMS among individuals with diabetes than among individuals without diabetes,^3,4,18^ our study focused on those with diabetes.

The survival advantage associated with BMS compared with treatment with GLP-1RAs may be explained by the greater relative decrease in BMI observed among patients who underwent BMS (−31.4%) compared with that achieved among patients treated with GLP-1RAs (−12.8%). Aminian et al^19^ showed an association of BMS with mortality during a mean follow-up of 4.9 years, while a minimum of 5% weight loss was necessary to reduce the risk for all-cause mortality among patients with diabetes who underwent BMS, compared with 20% among patients who did not undergo BMS. In the present study, participants who underwent BMS achieved a mean minimum weight loss more than the minimum threshold suggested by Aminian et al.^19^ This finding is compared with those who were treated with GLP-1RAs and achieved a mean minimum weight loss less than the threshold suggested for individuals who did not undergo BMS. The fact that the survival advantage disappeared after adjusting for maximal weight loss during follow-up suggests that the association between treatment type and all-cause mortality is mediated by the amount of weight loss.

In this study, BMS did not show a survival advantage compared with treatment with GLP-1RAs among patients with a diabetes duration of longer than 10 years, despite the long-term decrease in BMI. This finding may be explained by the adverse effects of prolonged diabetes duration, which masked the benefit associated with weight reduction. This result is in line with reports of greater prevention of diabetes complications after BMS among individuals with a shorter duration of diabetes.^14,15^ It is also possible that other factors related to aging could be mediating the association of BMS and GLP-1RA treatment with mortality. In addition, it could also be the case that the sample size of people with a diabetes duration of more than 10 years was too small to demonstrate a difference in the risk of mortality in this group. Additional studies are needed to establish the survival advantage associated with BMS compared with GLP-1RA treatment among patients with a diabetes duration of 10 years or less and to further understand why this advantage was not demonstrated among individuals with a diabetes duration of longer than 10 years.

Bariatric metabolic surgery^16^ and treatment with GLP-1RAs,^17^ separately, compared with standard of care, were each shown to reduce MACEs. Although the benefits associated with BMS and treatment with GLP-1RAs in reducing MACEs compared with standard of care have been well documented in recent years,^16,17^ a direct head-to-head prospective comparison between these 2 interventions has not been published, to our knowledge. Our finding of a comparable association with nonfatal MACEs between the 2 treatments supports the hypothesis that the association of treatment with GLP-1RAs with the incidence of MACEs is independent of weight loss. This idea is supported by the Harmony Outcomes study,^20^ which demonstrated a favorable association of GLP-1RA treatment with MACEs, although the difference in weight-loss changes between the GLP-1RA group and the control group was only 0.8 kg. Beyond its contribution to weight loss, a potential mechanism for the cardioprotective association of a high level of GLP-1RA is its capacity to reduce other risk factors associated with CVD, including hypertension, hyperlipidemia, and hyperglycemia. The anti-inflammatory associations of GLP-1RA treatment with stabilizing atherosclerosis plaque were also suggested as possible pathways for the observed contribution of treatment with GLP-1RAs in reducing CVD. Treatment with GLP-1RAs may also reduce myocardial apoptosis and oxidative stress, as well as increase myocardial glucose uptake and coronary blood flow.^21,22,23^ Although BMS increases native GLP-1, and treatment with GLP-1RAs increase GLP-1 analog levels, BMS increases the native GLP-1RAs mainly postprandially. In addition, several studies showed significantly higher postprandial GLP-1 levels after BMS among patients with sustained weight loss than among patients who regained weight, although fasting GLP-1 levels were similar and sometimes returned to a lower than normal value.^24,25^

An alternative possibility is that GLP-1RA administration offers a pharmacokinetic or pharmacodynamic advantage over the native GLP-1 levels that present after BMS, thus reducing the risk of MACEs. Although these hypotheses align with current knowledge, further studies are required to confirm the association and elucidate the underlying mechanism. In contrast to our findings, Stenberg and Näslund^13^ recently reported a lower risk for MACEs among individuals with diabetes who underwent BMS compared with treatment with GLP-1RAs. However, that study did not differentiate between individuals with and individuals without a history of CVD, mortality was assessed as 1 component of a composite outcome, data on body weight and weight change were missing in the GLP-1RA group, and most individuals included in the study underwent gastric bypass surgery, thus limiting the comparability of the results.

### Limitations

Our study has several limitations. First, the comparison groups may have differed in their characteristics due to the observational design. Therefore, major confounders were matched, followed by using multivariable models to account for additional potential confounders. Nevertheless, the possibility of residual confounding cannot be excluded, including the case that unmeasured differences between groups were present, such as the level of frailty of patients. In Israel, however, only minimal numbers of patients are ruled out for surgery within the public health care system, typically limited to severe medical conditions defined as contraindications.

Second, the attempt to strictly control for key potential confounders led to the exclusion of many eligible surgical patients. Third, the types of surgery and the types of GLP-1RAs were not considered in the analyses. In addition, the GLP-1RAs assessed in this study included only first-generation pharmacotherapies. Additional studies, with larger sample sizes and newer GLP-1RAs, are necessary to stratify by surgery type and by GLP-1RA type.

Fourth, the analysis did not consider the level of adherence to GLP-1RA treatment. Rather, an intention-to-treat approach was adopted, and patients were classified as treated with GLP-1RAs if they purchased GLP-1RAs for at least 6 months within a consecutive 12-month period during the study period. The association of adherence to treatment with outcomes should be considered in further studies.

Fifth, information regarding the cause of death was unavailable. This limitation is particularly important, given the lower mortality rates observed among patients who underwent BMS, despite no difference in the occurrence of MACEs. This finding raises the question as to which health conditions are less common among the patients undergoing BMS who are responsible for the lower mortality rates. A sensitivity analysis excluding patients with oncologic diagnoses partially addressed this limitation.

Sixth, while censoring patients when they switch treatment is essential for preserving the integrity of treatment comparisons, initiation of GLP-1RAs after BMS could be a signal that the BMS was not successful. This limitation should be acknowledged, considering that treatment switching is much more common in clinical data.

## Conclusions

This retrospective cohort study found that, among individuals with a diabetes duration of 10 years or less and without a prior history of CVD or congestive heart failure, BMS was associated with lower all-cause mortality compared with GLP-1RA treatment, over a median follow-up of 6.8 years. Weight reduction was identified as a mediating factor in this association. No difference between BMS and treatment with GLP-1RAs was observed in the risk of mortality among individuals with a longer duration of diabetes (>10 years), nor in the risk of MACEs among all patients.

## Notes

Supplement 1. **eMethods.** Baseline Measurements
eReference.
**eResults.** Results of Sensitivity Analysis **eTable.** Associations Between Treatment Type (BMS/GLP-1RA) and Mortality According to the Duration of Diabetes, Excluding Individuals With a Cancer Diagnosis in the First Two Years of Follow-Up **eFigure.** Selection of Patients Who Underwent BMS, and Matching to Patients Treated With GLP-1RAs

Supplement 2.
Data Sharing Statement
