# Supplementary material for: Bariatric Metabolic Surgery vs Glucagon-Like Peptide-1 Receptor Agonists and Mortality
Source: JAMA Netw Open. 2024 Jun 7;7(6):e2415392. doi: 10.1001/jamanetworkopen.2024.15392 (PMC11161844; doi:10.1001/jamanetworkopen.2024.15392)
Supplement: Supplement 1. — eMethods. Baseline Measurements eReference. eResults. Results of Sensitivity Analysis eTable. Associations Between Treatment Type (BMS/GLP-1RA) and Mortality According to the Duration of Diabetes, Excluding Individuals With a Cancer Diagnosis in the First Two Years of Follow-Up eFigure. Selection of Patients Who Underwent BMS, and Matching to Patients Treated With GLP-1RAs [file jamanetwopen-e2415392-s001.pdf]

Supplementary Online Content

Dicker D, Wolff Sagy Y, Ramot N, et al. Bariatric metabolic surgery vs glucagon-like peptide-1 receptor agonists and mortality. *JAMA Netw Open.* 2024;7(6):e2415392. doi:10.1001/jamanetworkopen.2024.15392

eMethods. Baseline Measurements
eReference.
eResults. Results of Sensitivity Analysis
eTable. Associations Between Treatment Type (BMS/GLP-1RA) and Mortality According to the Duration of Diabetes, Excluding Individuals With a Cancer Diagnosis in the First Two Years of Follow-Up
eFigure. Selection of Patients Who Underwent BMS, and Matching to Patients Treated With GLP-1RAs

This supplementary material has been provided by the authors to give readers additional information about their work.

© 2024 Dicker D et al. *JAMA Network Open.*

eMethods. Baseline Measurements

This observational, retrospective cohort study was based on data obtained from the electronic medical records of Clalit Health Services (Clalit). Clalit is the largest healthcare organization in Israel (country with mandatory universal health insurance for all citizens), providing care to 52% of the population (~4.8 million individuals). Different from other countries such as the United States, Clalit is both an insurer and a provider of health services. Clalit's information systems are fully digitized and contribute to a centralized data warehouse containing both administrative and clinical data.

The following variables were extracted as of the index date: sociodemographic variables including patient sex, age (years), ethnicity (Jewish/non-Jewish/unknown), socioeconomic status (SES; low, medium, high), immigration status (born in Israel, immigrated to Israel), and peripherality (1-10, from residence in the periphery to the center area). The Peripherality Index is based on the Israeli Central Bureau for Statistics' Peripherality Index of Local Authorities, which categorizes local authorities in Israel by their proximity to the central economic hub. It considers potential accessibility, which includes proximity to other local authorities and population size, as well as proximity to the Tel Aviv District.[1] Diagnoses of co-morbidities, including atrial fibrillation, hyperlipidemia, and hypertension, were defined based on any documentation prior to the index date. Laboratory data were assessed based on any documentation in the community setting. These data included HbA1c concentration; blood levels of fasting plasma glucose (mg/dL), total cholesterol (mg/dL), HDL cholesterol (mg/dL), LDL cholesterol (mg/dL), and triglycerides (mg/dL); systolic and diastolic blood pressure (mmHg); BMI (kg/m^2); and smoking status (former, current, or non-smoker). Pharmaceutical treatments (yes/no for each treatment) were

© 2024 Dicker D et al. *JAMA Network Open.*

assessed based on ≥1 filled prescription during the year before the index date. These treatments included beta blocking agents, calcium channel blockers, agents acting on the renin-angiotensin system, lipid modifying agents, insulins, non-insulin injectable therapy, blood glucose lowering drugs, antidepressants, medications for weight reduction, and Sodium Glucose co-Transporter 2 (SGLT-2) Inhibitors.

© 2024 Dicker D et al. *JAMA Network Open.*

eReference.

1. Tsibel CBoSSMD-N. Peripherality Index of Local Authorities in Israel: Combination of Potential Accessibility Index with Proximity to the Tel Aviv District. 2009;doi:https://www.cbs.gov.il/he/publications/DocLib/pw/pw45/pw45.pdf

© 2024 Dicker D et al. *JAMA Network Open.*

eResults. Results of Sensitivity Analysis

A sensitivity analysis was conducted for the primary outcome (mortality), while excluding from the analysis individuals with a cancer diagnosis in the first two years of follow-up, yielded results consistent with those of the main analysis, (adjusted HR: 0.33, 95% CI: 0.21-0.52). The association was preserved when the percentages of the maximal change in HbA1c concentration during follow up was included in the model (HR: 0.36, 95%CI: 0.22-0.58), but disappeared once the percentages of the maximal change in BMI level was also introduced into the model (HR: 0.58, 95%CI: 0.32-1.03) (eTable).

© 2024 Dicker D et al. *JAMA Network Open.*

eTable. Associations Between Treatment Type (BMS/GLP-1RA) and Mortality According to the Duration of Diabetes, Excluding Individuals With a Cancer Diagnosis in the First Two Years of Follow-Up

**Model**	**≤10 y with diabetes n=4,683**	**>10 y with diabetes n=1,302**
**Hazard ratios (95% CI) for mortality (BMS versus treatment with GLP-1RA)**		
Unadjusted	**0.30 (0.20-0.43)**	**0.59 (0.37-0.93)**
Adjusted[a]	**0.33 (0.21-0.52)**	0.60 (0.35-1.03)
Adjusted, including HbA1c level maximal change	**0.36 (0.22-0.58)**	NA
Adjusted, including BMI level maximal change	0.58 (0.32-1.03)	NA

[a] The models were adjusted for the following variables as of the index date: diabetes duration (months), age (years), ethnicity (Arabs, Jews), BMI (kg/m^2), HbA1c concentration, socioeconomic status, diagnosis of atrial fibrillation, diagnosis of hyperlipidemia, diagnosis of hypertension, smoking status (non-smoker, former, current, unknown), and using agents acting on the renin-angiotensin system, lipid modifying agents, insulin, SGLT-2 inhibitors, and other blood glucose-lowering drugs. No multicollinearity was observed between variables included in the analysis (VIFs<1.3). The models met the proportional hazards assumption.

© 2024 Dicker D et al. *JAMA Network Open.*

eFigure. Selection of Patients Who Underwent BMS, and Matching to Patients Treated With GLP-1RAs

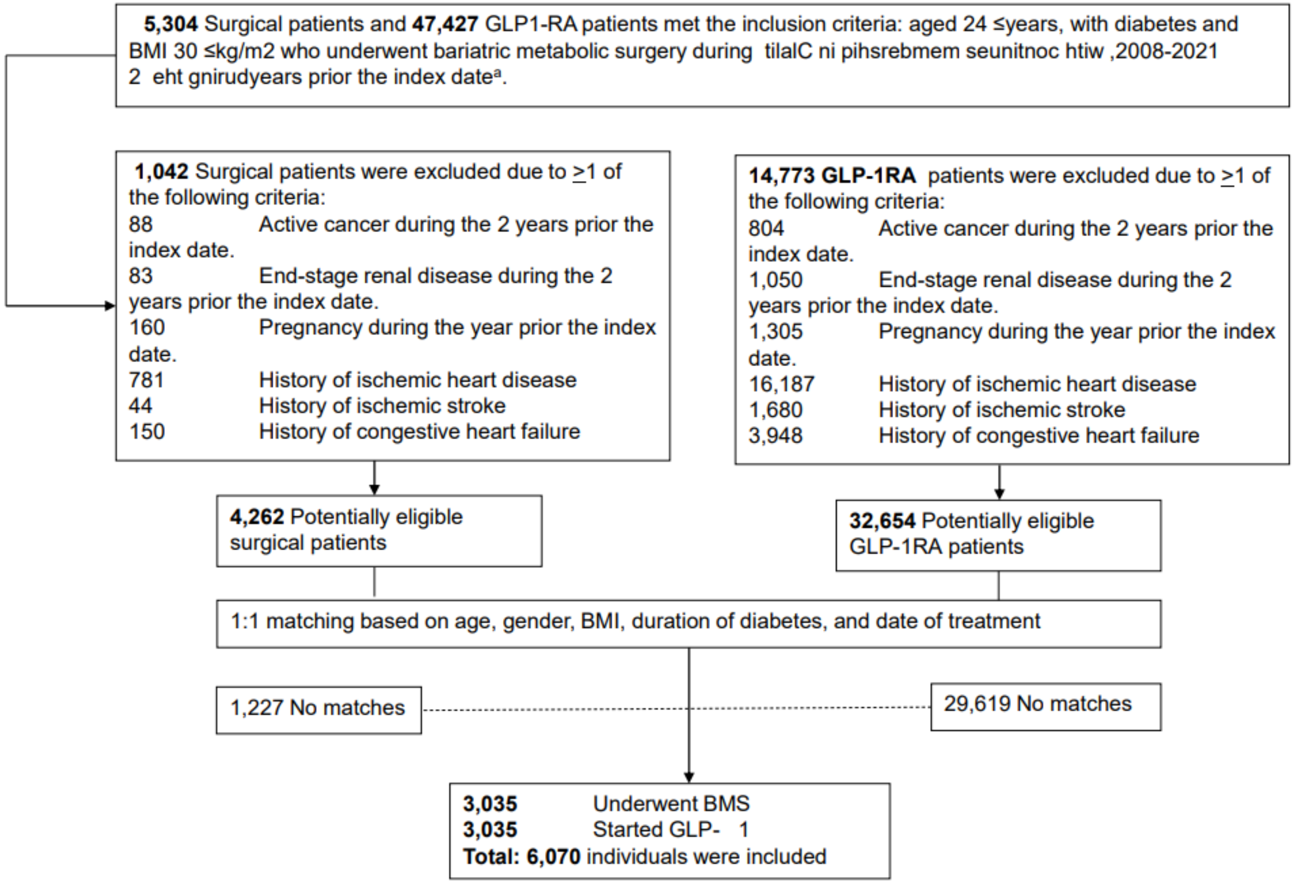

© 2024 Dicker D et al. *JAMA Network Open.*
