# Supplementary material for: Bariatric Metabolic Surgery vs Glucagon-Like Peptide-1 Receptor Agonists and Mortality
Source: JAMA Netw Open. 2024 Jun 7;7(6):e2415392. doi: 10.1001/jamanetworkopen.2024.15392 (PMC11161844; doi:10.1001/jamanetworkopen.2024.15392)
Supplement: Supplement 2. — Data Sharing Statement [file jamanetwopen-e2415392-s002.pdf]

Data Sharing Statement

Dicker. Bariatric Metabolic Surgery vs Glucagon-Like Peptide-1 Receptor Agonists and Mortality. *JAMA Netw Open*. Published June 07, 2024.
doi:10.1001/jamanetworkopen.2024.15392

Data
Data available: No

Additional Information
Explanation for why data not available: The data are not publicly available due to privacy restrictions.
